# Impact of Dose Perturbations Around Brachytherapy Seeds in External-Beam Radiotherapy Planning: A Fundamental and Clinical Validation Using Treatment Planning System-Based Monte Carlo Simulations

**DOI:** 10.7759/cureus.48041

**Published:** 2023-10-31

**Authors:** Shuta Ogawa, Keisuke Yasui, Naoki Hayashi, Yasunori Saito, Shinya Hayashi

**Affiliations:** 1 Department of Radiation Oncology, Tohoku University Graduate School of Medicine, Sendai, JPN; 2 Department of Radiation Physics and Technology, Southern Tohoku Proton Therapy Center, Koriyama, JPN; 3 School of Medical Sciences, Fujita Health University, Toyoake, JPN; 4 Division of Radiology, Fujita Health University Hospital, Toyoake, JPN; 5 Department of Radiation Oncology, Fujita Health University School of Medicine, Toyoake, JPN

**Keywords:** radiotherapy, brachytherapy seed, treatment planning systems, low-dose rate brachytherapy, monte carlo simulation

## Abstract

Background

This study evaluates dose perturbations caused by nonradioactive seeds in clinical cases by employing treatment planning system-based Monte Carlo (TPS-MC) simulation.

Methodology

We investigated dose perturbation using a water-equivalent phantom and 20 clinical cases of prostate cancer (10 cases with seeds and 10 cases without seeds) treated at Fujita Health University Hospital, Japan. First, dose calculations for a simple geometry were performed using the RayStation MC algorithm for a water-equivalent phantom with and without a seed. TPS-independent Monte Carlo (full-MC) simulations and film measurements were conducted to verify the accuracy of TPS-MC simulation. Subsequently, dose calculations using TPS-MC were performed on CT images of clinical cases of prostate cancer with and without seeds, and the dose distributions were compared.

Results

In clinical cases, dose calculations using MC simulations revealed hotspots around the seeds. However, the size of the hotspot was not correlated with the number of seeds. The maximum difference in dose perturbation between TPS-MC simulations and film measurements was 3.9%, whereas that between TPS-MC simulations and full-MC simulations was 3.7%. The dose error of TPS-MC was negligible for multiple beams or rotational irradiation.

Conclusions

Hotspots were observed in dose calculations using TPS-MC performed on CT images of clinical cases with seeds. The dose calculation accuracy around the seeds using TPS-MC simulations was comparable to that of film measurements and full-MC simulations, with differences within 3.9%. Although the clinical impact of hotspots occurring around the seeds is minimal, utilizing MC simulations on TPSs can be beneficial to verify their presence.

## Introduction

Two types of photon-beam radiotherapy are available for prostate cancer: external-beam radiotherapy (EBRT) and brachytherapy. Low-dose-rate (LDR) brachytherapy uses permanent radioactive seeds implanted into the prostate. It is an excellent option for low and selected intermediate-risk localized prostate cancer. However, given the nature of brachytherapy, where the delivered dose can be substantially less than the planned dose, recurrence rates are higher than they should be [1]. Although there are various options for salvage therapy for locally recurrent prostate cancer after primary low-dose-rate prostate brachytherapy, salvage therapy with EBRT is being considered for several reasons such as non-invasive treatment and high availability [2]. The seeds used in LDR brachytherapy are typically high-atomic-number (high-Z) materials [3], and high-Z materials cause dose perturbations [4-8]. Steinman et al. evaluated the dose perturbation around the seed under simple geometry using film and Monte Carlo (MC) simulation [4]. Chow and Grigorov reported on the evaluation of dose perturbation around the seeds using MC simulation and indicated that the dose perturbation may have a clinical impact [5]. However, these studies were conducted under clinical conditions using CT imaging; therefore, the precise impact of dose perturbations around the seeds within the patient’s body has not been evaluated.

The dose simulation within the patient’s body is typically conducted using a treatment planning system (TPS). The dose-calculation algorithm commonly employed in TPSs, known as convolution/superposition methods, cannot accurately calculate the backscatter and attenuation of photon beams around high-Z materials. In contrast, MC simulations can accurately calculate doses, including around high-Z materials, in a trade-off for a longer computation time. Studies have reported accurate dose calculations near extremely small high-Z materials such as seeds using MC algorithms [4,5,9]. However, the doses from MC simulations in these reports were calculated using MC simulation tools independent of the TPSs, making it difficult to replicate simulations at all facilities. With the development of faster MC algorithms and improvements in computer performance in recent years, MC simulations on TPSs are now widely performed. RayStation (RaySearch Laboratories, version 10A), a general-purpose TPS, is equipped with MC calculations that are highly accurate [10-13]. Richmond et al. reported that the RayStation MC dose calculation algorithm is sufficiently accurate in a range of testing geometries and is suitable for clinical use [12]. The feature has enabled the confirmation of dose perturbations in the prostate with seeds using TPS-based MC (TPS-MC) simulations even in general clinical practice; however, it has not been investigated so far.

Hence, this study aims to evaluate dose perturbation caused by non-radioactive seeds in clinical cases using TPS-MC.

## Materials and methods

Seed implants

**Figure 1:**
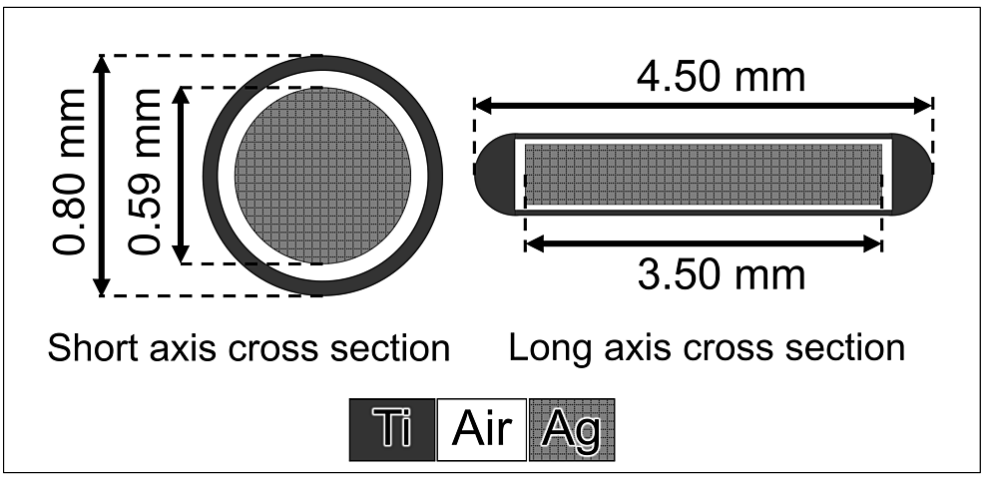
Schematic diagram showing the dimensions of the TheraAgX 100 (AgX). 125-I was adsorbed chemically onto the silver surface of the AgX. Ti: titanium; Ag: silver

We used nonradioactive TheraAgX100 (AgX; Theragenics Corporation, Buford, GA, USA) seeds for film measurement, commonly used in iodin-125 (125-I)-inserted brachytherapy. AgX contains a cylindrical silver rod (3.50 mm in length and 0.59 mm in diameter) within a titanium capsule measuring 4.50 mm, 0.80 mm, and 0.05 mm in the outer length, outer diameter, and wall thickness, respectively (Figure 1) [14]. The mass densities of the titanium and silver were 4.54 g/cm^3^ and 10.49 g/cm^3^, respectively. Furthermore, 125-I was chemically adsorbed onto the silver surface of the AgX. Full-MC simulations can be used to model the complex geometry of AgX. In contrast, it is not possible to model AgX in TPS because of its inability to accurately represent small structures on the order of 0.1 mm. Therefore, we used titanium (mass density: 4.54 g/cm^3^) of the same size as the AgX in our model on the TPS.

MC simulations

The MC dose engine used in RayStation is the same fluence computation in the linear accelerator head as in the collapsed cone dose engine. In addition to measurement-based validation, each TPS-MC simulation of the clinical cases was cross-checked against a full-MC dose engine (EGSnrc) [15]. The dose used in the TPS-MC simulation was calculated as a 0.1% average statistical uncertainty in voxels, with more than 50% of the maximum dose.

The particle and heavy ion transport code system (PHITS, version 3.11) was used as the full-MC dose engine [16]. Phase-space files (PSFs) were used to simulate conventional with flattening filter (WFF) beams. The PSFs for the 6 MV and 10 MV photon beams were provided by Varian Medical Systems (Palo Alto, CA, USA). These PSFs store particle information tallied on a plane proximately upstream of the movable jaws. The cutoff energies for the photons and electrons were set to 0.01 MeV. The number of photon histories in full-MC was 5.0 × 10^11^. The physics list and any other pertinent parameters that could affect MC simulations were set to their default values in PHITS [17]. To observe any potential dose perturbations around the seed, the full-MC calculation grid size was set to 0.8 mm, corresponding to the diameter of the seed. The dose used in the full-MC simulations was calculated to be 1% of the average statistical uncertainty.

Investigation under simple geometry

**Figure 2:**
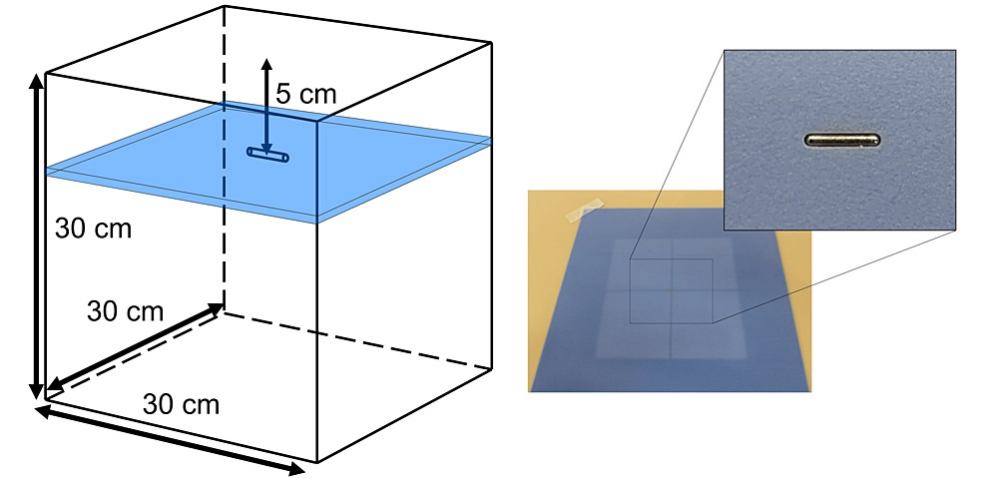
Geometric scheme of a simple condition. One seed was placed at a depth of 5 cm on the beam axis. A water-equivalent phantom with the same thickness as the seed was created to fill the air gap around the seed.

TPS-MC and full-MC calculations and film measurement with a nonradioactive seed were conducted under a simple geometry, as shown in Figure 2. The seed was inserted into a water-equivalent phantom (Tough water; Kyoto Kagaku Co., Ltd., Kyoto, Japan) and scanned using a Toshiba Aquilion LB CT scanner (Toshiba Medical Systems Corporation, Otawara, Japan) in 0.5 mm slice thickness. For comparison with the MC calculation, the dose using collapsed cone convolution (CCC) was also calculated. Both TPS calculated doses of 6 MV and 10 MV WFF beams with a dose grid size of 1 × 1 × 1 mm^3^. Furthermore, the field size was set to 10 × 10 cm^2^ using movable jaws at the isocenter. Percentage depth doses (PDDs) were calculated using each dose-calculation algorithm with and without a seed. The PDD output from the TPS was interpolated at 0.1 mm intervals on the TPS. Moreover, the PDDs without seeds calculated via MC simulations were compared with those calculated via CCC using the gamma index metric [18].

Film measurements using a radiochromic film (Gafchromic EBT3 film; Ashland Specialty Ingredients, NJ, USA) were conducted to verify the accuracy of each dose-calculation algorithm. Film measurements were performed according to a standard film measurement protocol and under the same conditions as the MC simulations [19]. The films were positioned at three locations upstream from the seed, spaced at 0.8 mm intervals, and at three locations downstream. Furthermore, films were placed at two additional positions, 5 mm and 8 mm downstream from the seed. All films were analyzed using a flatbed scanner (Epson Scanner ES10000G; Seiko-Epson Corporation, Nagano, Japan) and ImageJ software (version 1.52; National Institutes of Health, Bethesda, MD, USA), and the difference in relative dose between cases with and without the seed was measured [20,21]. All films were processed after irradiation for at least 24 hours and analyzed by red channel. To obtain dose calibration curves, the same lot films were exposed at the dose levels of 0, 25, 50, 75, 100, 150, 200, 250, 300, and 400 monitor units. The analysis area of the film was set on the beam axis according to the seed size as in the MC simulation.

Investigation using clinical cases

We conducted a retrospective analysis of treatment planning data for prostate cancer, approved by the Institutional Review Board (IRB) of Fujita Health University, Japan. The analysis included 10 cases with seeds and 10 cases without seeds. Dose distributions in clinical cases were recalculated using the CCC and TPS-MC algorithms. For the seeded cases, 35-69 seeds were used, depending on the prostate volume. Using the CCC algorithm, the prescription dose to 95% of the planning target volume (PTV) was set to 78 Gy/39 fractions for all treatment plans. The prescribed dose was set to the same value to compare cases with and without the seed directly. The beam energy was set to 10 MV, and volumetric modulated arc therapy (VMAT) was used as the irradiation technique in all cases. The plan calculated by the CCC algorithm was used as the reference plan, the same fluence and monitor units were used for both calculations, and the grid size for the dose calculation was set to 2 mm. The region of interest was created for all inserted seeds on the planning CT images and defined as titanium. The planning CT images included metallic artifacts due to the seeds because metallic artifact reduction techniques were not used. To eliminate the artifacts, the PTV outside the region defined as titanium was defined as water.

Dosimetric comparisons and evaluations performed for both CCC and the TPS-MC algorithm included D_99%_ (isodose that covers 99% of PTV), D_98%_, D_95%_, D_50%_, D_2%_, and D_0.03 cc_ (the highest dose to 0.03 cm^3^ of PTV), Paddick’s conformity index (CI), and homogeneity index (HI) [22,23]. Paddick’s CI was calculated as follows:

$CI = \frac{{TV_{PIV}}^{2}}{TV\times V_{PIV}}$

where TV is the target volume, TV_PIV_ is the target volume covered by the prescribed isodose volume (PIV), and V_PIV_ is the total PIV. CI has an ideal value of one and plan quality decreases with decreasing index value. HI was calculated as follows:

$HI = \frac{D_{2\%}-D_{98\%}}{D_{p}}$

where D_p_ is the prescription dose. The lower (closer to zero) HI, the better the dose homogeneity. The dose differences of D_99%_, D_98%_, D_95%_, D_50%_, D_2%_, and D_0.03 cc_ in cases with and without seeds were evaluated using a two-sided parametric Student’s t-test for paired samples. The dose difference was calculated as the difference between the dose indices by dose calculations using TPS-MC and those using the CCC algorithm. Additionally, CIs and HIs were evaluated for each case, both with and without seeds, comparing cases calculated with CCC and TPS-MC using a two-sided parametric Student’s t-test for paired samples.

## Results

Dose perturbations under simple geometry

**Figure 3:**
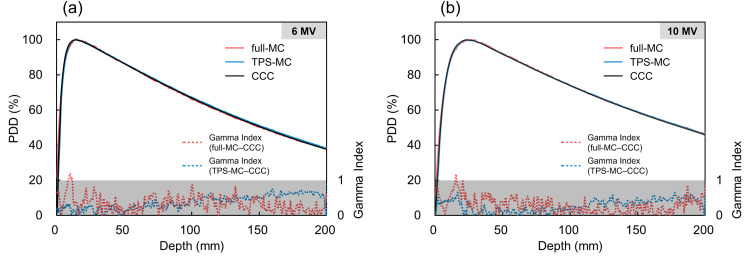
PDDs without the seed calculated via CCC, TPS-MC simulation, and full-MC simulation. The differences in PDD were less than 1% and the gamma index passing rate (using 1%/1 mm criteria) was 100% at 6 MV and 10 MV for all depths beyond the build-up region. Gray areas indicate regions where the gamma index is less than 1. PDD: percentage depth dose; CCC: collapsed cone convolution; TPS-MC: treatment planning system-based Monte Carlo; full-MC: treatment planning system-independent Monte Carlo

Figure 3 shows the comparisons of the PDDs without the seed calculated via CCC, TPS-MC simulations, and full-MC simulations. The differences in PDD were less than 1%, and the gamma index passing rate (using 1%/1 mm criteria) was 100% at 6 MV and 10 MV for all depths beyond the build-up region.

**Figure 4:**
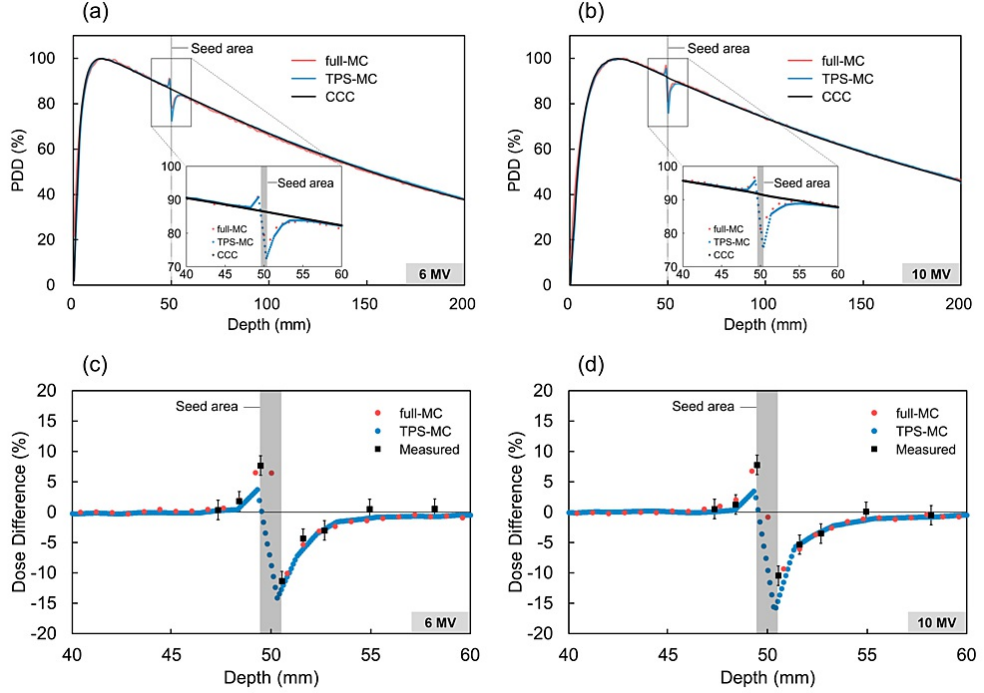
(a) and (b) PDDs with the seed. (c) and (d) The difference between PDDs, with and without a seed, calculated via MC simulations and measured by films. Dose enhancement was observed in the upstream region of the seed, whereas dose reduction was observed in the downstream region. The dose difference between the full-MC simulations and film measurements was in good agreement, with differences within 1.8%. The difference in the dose perturbations between the TPS-MC and film measurements was more pronounced at positions adjacent to the seed. PDD: percentage depth dose; CCC: collapsed cone convolution; TPS-MC: treatment planning system-based Monte Carlo; full-MC: treatment planning system-independent Monte Carlo

Figure 4a and Figure 4b show PDDs with the seed. Figure 4c and Figure 4d show the difference between the PDDs with and without a seed calculated via MC simulations and measured using films. The film measurement includes a 3.2% uncertainty in the measurement of the red channel [24]. The area of dose perturbation under simple geometry ranged from 2 mm upstream to 5 mm downstream. Furthermore, the maximum difference in dose perturbations between the TPS-MC simulations and film measurements was 3.9% (10 MV, downstream from the seed) at locations adjacent to the seed. The difference between the full-MC simulations and film measurements was 1.8% (6 MV, downstream from the seed). There was no dependence of the difference in dose perturbations between MC simulations and film measurements on beam energy. Of note, CCC could not calculate dose perturbations due to the seed.

Dose perturbations in clinical cases

**Figure 5:**
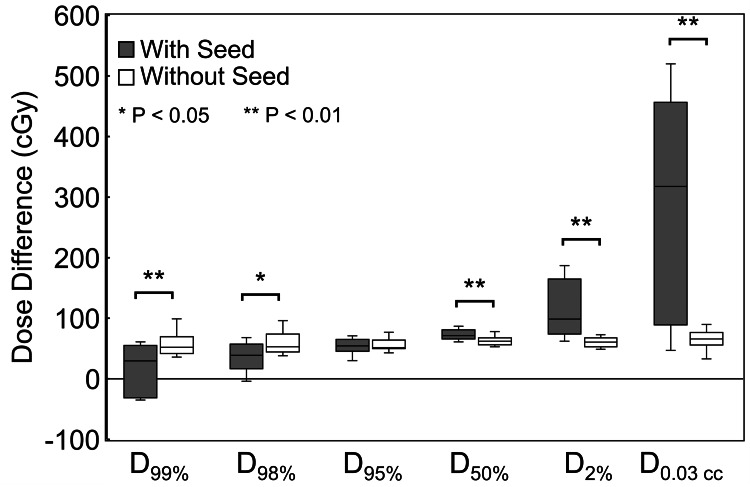
Differences in dose indices in clinical cases with and without seeds. The dose difference was calculated as the difference between the dose indices by dose calculations using TPS-MC and those using the CCC algorithm. The dose calculated using TPS-MC simulations was slightly higher than that calculated using the CCC. In this study, the prescribed dose was determined based on the CCC dose calculation. CCC: collapsed cone convolution; TPS-MC: treatment planning system-based Monte Carlo; full-MC: treatment planning system-independent Monte Carlo; D_x%_: isodose that covers x% of the planning target volume; D_0.03 cc_: the highest dose to 0.03 cm^3^ of the planning target volume

Figure 5 shows the difference in dose indices between the TPS-MC simulation and the CCC algorithm in clinical cases with and without seeds. The dose difference was calculated as the difference between the dose indices by dose calculations using TPS-MC and those using the CCC algorithm. The dose calculated using TPS-MC simulations was slightly higher than that calculated using CCC. D_99%_ and D_98%_ were significantly lower in the seeds (D_99%_: p < 0.01, D_98%_: p < 0.05). D_95%_ was comparable for cases with and without seeds, whereas D_50%_, D_2%_, and D_0.03 cc_ were significantly higher in seeds (D_50%_, p < 0.01; D_2%_, p < 0.01; D_0.03 cc_, p < 0.01). The difference in D_0.03 cc_ between TPS-MC simulations and CCC averaged 284 cGy with seeds, 216 cGy higher than without seeds. Furthermore, the difference in the dose indices tended to be larger in the high-dose area in cases with seeds.

**Figure 6:**
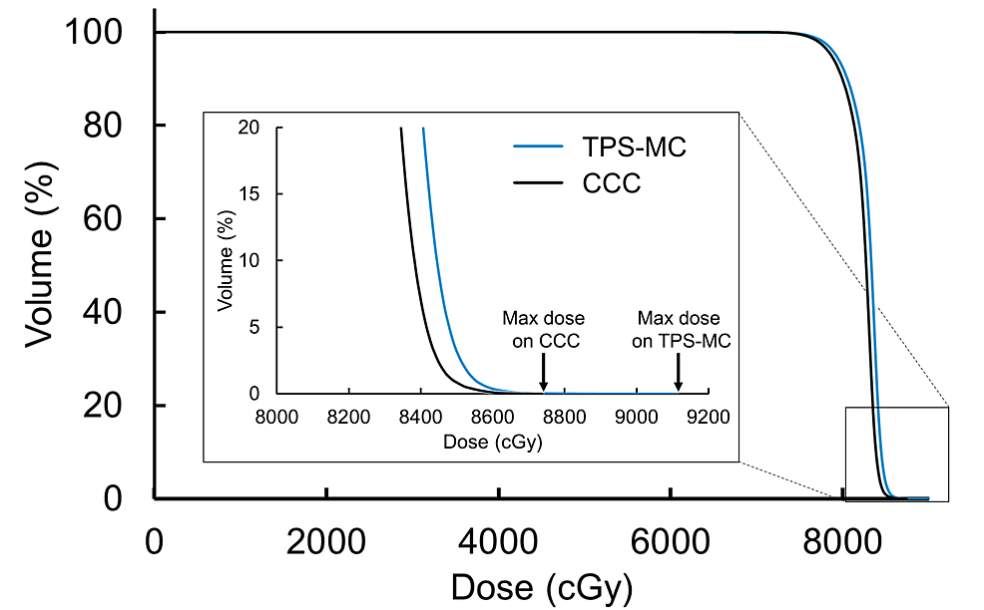
DVH of PTV calculated using TPS-MC simulation and CCC in one clinical case with seeds. Hotspots around the seeds are observed exclusively in dose calculations using TPS-MC. DVH: dose-volume histogram; PTV: planning target volume; CCC: collapsed cone convolution; TPS-MC: treatment planning system-based Monte Carlo

**Figure 7:**
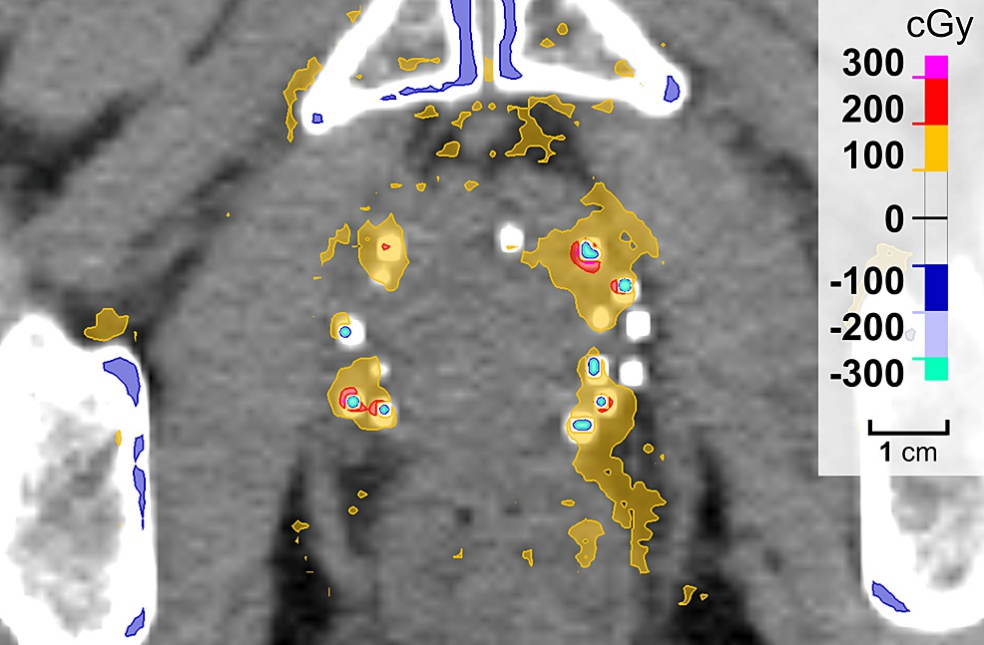
Distribution of the dose differences in one clinical case with seeds. The high-dose areas occurred around the seeds, whereas the doses decreased in the areas defined as the seeds.

Figure 6 and Figure 7 show the dose-volume histogram (DVH) of the PTV and the distribution of dose differences, respectively, in the cases with seeds. The dose distribution calculated using TPS-MC simulations showed a high-dose region around the seeds in the PTV. Moreover, regardless of the dose-calculation algorithm, the high-dose region was not observed in the cases without seeds.

**Figure 8:**
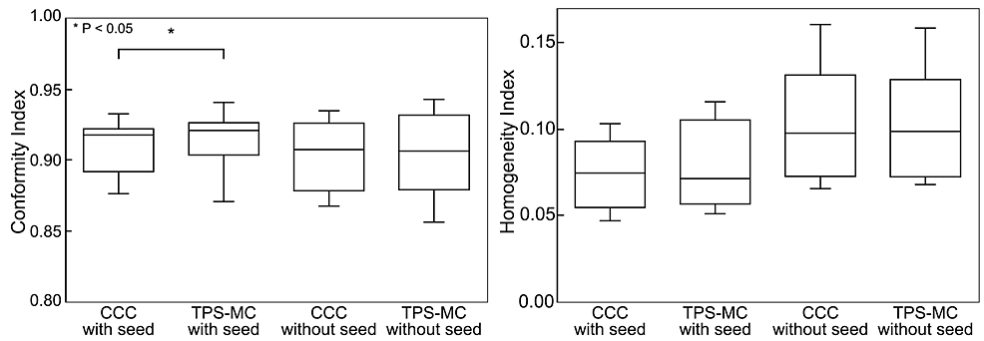
CIs and HIs of the clinical cases. The dose conformity to PTV using TPS-MC simulations was higher than that using CCC in cases with seeds (p < 0.05). No statistically significant differences were observed in dose homogeneity in the PTV. CI: conformity index; HI: homogeneity index; PTV: planning target volume; CCC: collapsed cone convolution; TPS-MC: treatment planning system-based Monte Carlo

Figure 8 shows the CIs and HIs calculated for the clinical cases. The dose conformity to the PTV using TPS-MC was higher than that obtained using CCC in cases with seeds (p < 0.05). However, this trend was not observed in the cases without seeds. Furthermore, the range of CIs, with and without seeds, was wider in cases calculated with TPS-MC simulations than in those with CCC. No statistically significant differences were observed in dose homogeneity in the PTV, which depended on the dose-calculation algorithm. The details of each clinical case are summarized in Table 1. The dose differences of D_0.03 cc_, CI, and HI were not correlated with the number of seeds.

**Table 1:** Details of each clinical case including irradiation technique, beam energy, the number of seeds, dose difference of D0.03 cc, CI, and HI. VMAT: volumetric modulated arc therapy; CCC: collapsed cone convolution; TPS-MC: treatment planning system-based Monte Carlo; D_0.03 cc_: the highest dose to 0.03 cm^3^ of the planning target volume; CI: conformity index; HI: homogeneity index

	Patient number	Irradiation technique	Beam energy (MV)	Number of seeds	Dose difference of D_0.03 cc _(cGy)	CI		HI	
						CCC	TPS-MC	CCC	TPS-MC
With seed	1	VMAT	10	44	47	0.916	0.923	0.103	0.103
	2	VMAT	10	69	96	0.933	0.941	0.054	0.057
	3	VMAT	10	44	441	0.895	0.908	0.06	0.076
	4	VMAT	10	44	371	0.905	0.917	0.094	0.111
	5	VMAT	10	37	287	0.92	0.924	0.057	0.061
	6	VMAT	10	54	348	0.92	0.919	0.082	0.104
	7	VMAT	10	37	160	0.876	0.871	0.047	0.051
	8	VMAT	10	50	520	0.928	0.927	0.047	0.057
	9	VMAT	10	50	68	0.919	0.926	0.067	0.067
	10	VMAT	10	40	502	0.881	0.89	0.093	0.116
Without seed	1	VMAT	10	-	33	0.923	0.928	0.071	0.071
	2	VMAT	10	-	53	0.913	0.913	0.097	0.097
	3	VMAT	10	-	115	0.868	0.856	0.161	0.159
	4	VMAT	10	-	57	0.879	0.88	0.099	0.101
	5	VMAT	10	-	70	0.935	0.942	0.073	0.073
	6	VMAT	10	-	64	0.908	0.905	0.092	0.093
	7	VMAT	10	-	72	0.876	0.883	0.124	0.123
	8	VMAT	10	-	90	0.882	0.877	0.154	0.148
	9	VMAT	10	-	68	0.907	0.908	0.113	0.112
	10	VMAT	10	-	60	0.935	0.943	0.066	0.068
With seed			Mean	47	284	0.909	0.915	0.07	0.08
			Std		180	0.0195	0.0203	0.0209	0.0254
Without seed			Mean	-	68	0.903	0.904	0.105	0.105
			Std		22	0.0249	0.0292	0.0332	0.0315

## Discussion

Our results indicated that dose perturbation around the seed can be accurately calculated using TPS-MC on CT images of clinical cases with seeds. These findings suggest that TPS-MC simulations may serve as a valuable means to confirm the dose perturbation around the seed.

Doses around the seeds were consistent within 3.9% for the TPS-MC simulations, full-MC simulations, and film measurements. In particular, the difference was substantial at measurement locations adjacent to the seed. This may be attributed to the differences in the dose-calculation models, the uncertainty of film dosimetry, and simplifications assumed by TPS-MC simulations in terms of physical processes [19,24]. Certain reports on dose calculations in TPSs have shown that they can be performed accurately, including around high-Z materials [25,26]. However, these reports were based on large-volume high-Z materials and not on extremely small high-Z materials, such as the seeds used in this study. The inconsistent dose perturbation at locations adjacent to the seeds can be attributed to the differences in the seed modeling and the seed shape not being stable owing to the CT artifacts. The seeds in TPS-MC simulations were modeled with only titanium, whereas those in full-MC simulations were modeled with titanium and silver. Therefore, a difference was observed in the atomic number of the seeds, which may have caused errors in the calculated doses. In addition, high-Z materials produce beam-hardening artifacts and CT number errors, which result in partial image loss and geometry errors in CT images used for treatment planning. Considering these uncertainties, the dose error of the TPS-MC simulations was shown to be within a few percent for a single beam and negligible for multiple beams or rotational irradiation.

High-dose areas were observed around the seed in the dose distribution calculated by TPS-MC simulations for a patient with seeds. This was because of the dose perturbation observed under a simple geometry. The dose increased and decreased under simple geometry, and only extraordinary dose increases were observed in clinical cases. This may be because of the lateral scattering by the high-Z material, which contributed to the dose perturbation. Moreover, the dose increase was a localized effect and resulted in remarkable changes in the indices assessing the hotspots, such as D_0.03 cc_. The difference in D_0.03 cc_ between TPS-MC simulations and CCC was significant when the inserted seeds were close to each other. In contrast, the local dose increase was confined to the PTV and had a negligible clinical impact as the area around the seed received the dose by brachytherapy. While a minor clinical impact of the hotspots may occur around the urethra, the TPS-MC simulation was shown to be a valuable tool for confirming these hotspots around the urethra.

Dose calculations using TPS-MC simulations in clinical cases with seeds showed significantly higher CIs. The increase in CI was because of dose perturbation around the seeds, which decreased the areas not covered by the prescribed dose in the TV and increased TV_PIV_. However, an increase in CI to that extent would not have a clinical impact.

No remarkable difference was observed in HI between TPS-MC simulations and CCC. D_2%_ was used as a representative value of the high-dose area for the HI calculations. The results from the clinical cases indicate that the high-dose area that considerably disturbed the dose homogeneity within the PTV was a dose area higher than D_2%_. Therefore, disturbances in dose homogeneity within the PTV may not have been adequately evaluated by the HI used in this study.

Stereotactic body radiation therapy (SBRT), one of the treatment options for localized prostate cancer, requires the placement of fiducial markers before radiation therapy to account for prostate motion during treatment and to prevent over-irradiation of normal surrounding tissue [27,28]. The fiducial markers are classified as high-Z material as well as the seed and may cause similar dose perturbations. Therefore, treatment planning for SBRT may need to take into account the possibility of a 2% to 3% dose increase in the prescribed dose.

This study contained several limitations. First, we could not accurately define each seed used in the clinical cases. Owing to the insertion of 35-69 seeds into the prostate during brachytherapy, it was not feasible to define all the seeds of titanium. Therefore, seeds were defined based on CT values, which caused uncertainty in the seed sizes owing to artifacts in the CT images. Regarding the impact of seed size uncertainty, this study evaluated the results by comparing the TPS-MC and CCC calculations using the same data; therefore, the impact of seed size uncertainty on the results is considered to be negligible. However, seed size affects the degree of dose perturbation; hence, it should be considered when more accurate verification is required. Second, the results presented in this study are derived from dose calculations using the MC simulation in RayStation. It should be noted that using MC simulation in a different TPS may lead to different results. Additionally, it should also be noted that the hotspots vary due to the inter-fractional seed position difference.

## Conclusions

We investigated the dose perturbation around seeds based on MC simulations and film measurements. The accuracy of dose calculations around the seed under simple geometry using TPS-MC simulations was comparable to that of film measurements and full-MC simulations, with differences within 3.9%. Furthermore, the dose error around the seeds in TPS-MC simulations was negligible for multiple beams or rotational irradiation. In the clinical cases, dose calculations using MC simulations revealed hotspots around the seeds. While a minor clinical impact of the hotspots may occur, the TPS-MC simulation was shown to be a valuable tool for confirming these hotspots.
